# Modeling of affective-negative content in the course of schizophrenia

**DOI:** 10.1192/j.eurpsy.2022.799

**Published:** 2022-09-01

**Authors:** A. Barkhatova, O. Kolyago

**Affiliations:** Mental Health Research Center, Department Of Endogenous Mental Disorders And Affective Conditions, Moscow, Russian Federation

**Keywords:** Depression, negative symptoms, schizophrenia, abulia

## Abstract

**Introduction:**

The world literature presents ambiguous results regarding the conjugation of negative and depressive syndromes, due to an incomplete understanding of the main symptoms of depression in schizophrenia.

**Objectives:**

To analyze the variants of the conjugation of depressive and negative symptoms at different stages of schizophrenia.

**Methods:**

We used the data of our own observations (238 patients with a diagnosis of schizophrenia and no more than 5 years of experience of the disease) and compared them with the previously published results of studies. As a hypothesis, we analyze the variants of the conjugacy of affect and the negative domain within the framework of a single discrete field of schizophrenia.

**Results:**

The analysis shows that with the apparent heterogeneity of the psychopathological structure, some depressive features, such as apathy, anhedonia and social autism (characteristic of negative symptoms), tend to the abulia factor, whereas low mood, suicidal thoughts, pessimism, show affinity for the cluster of impoverishment of expression, that is, they represent an attenuated type of negative symptoms tending to the affective spectrum.

**Conclusions:**

The conjugacy of depressive and negative disorders in schizophrenia, taking into account their phenomenological similarity, allows us to formulate a hypothesis about their existence within the framework of a single continuum model. The proposed continuum model can be used to understand the processes underlying pathogenesis and formulate the principles of personalized treatment and can be used as a starting point for research on the underlying biological processes and personalized treatment.

**Disclosure:**

No significant relationships.

